# PROTOCOL: The effectiveness of abstinence‐based and harm reduction‐based interventions in reducing problematic substance use in adults who are experiencing severe and multiple disadvantage homelessness: A systematic review and meta‐analysis

**DOI:** 10.1002/cl2.1246

**Published:** 2022-07-07

**Authors:** Chris O'Leary, Rob Ralphs, Jennifer Stevenson, Andrew Smith, Jordan Harrison, Zsolt Kiss

**Affiliations:** ^1^ Policy Evaluation and Research Unit Manchester Metropolitan University Manchester UK; ^2^ Substance Use and Associated Behaviours Research Unit Manchester Metropolitan University Manchester UK; ^3^ Independent consultant London UK; ^4^ ZK Analytics Oxford UK

## Abstract

**Background:**

Homelessness is a major social and public health concern. It is a traumatic experience, and can have a devastating effect on those experiencing it. People who are homeless often face significant barriers when accessing public services, and those experiencing more visible and extreme forms of homelessness have often faced adverse childhood events, extreme social disadvantage, physical, emotional and sexual abuse, neglect, low self‐esteem, poor physical and mental health, and much lower life expectancy compared to the general population. Problematic substance use is disproportionately high amongst people experiencing homelessness, with many using drugs and alcohol to deal with the stress of living on the street, to keep warm, or to block out memories of previous abuse or trauma. Drug overdose is a major cause of death for people experiencing street homelessness. Substance dependency can also create barriers to successful transition to stable housing. There is ongoing policy interest in the effectiveness of different interventions that aim to stop, reduce or prevent problematic substance use, and there is specific interest in the relative effectiveness of interventions that adopt harm reduction or abstinence‐based approaches.

**Objectives:**

The objective of this review is to understand the effectiveness of different substance use interventions. The review will consider the effectiveness of harm reduction‐based interventions, and abstinence‐based interventions, for adults experiencing homelessness. The focus of the review is on high‐income countries.

**Search Methods:**

The primary source of studies for potential inclusion in this review is the Homelessness Effectiveness Studies Evidence and Gaps Maps (EGM). The first of these was published in 2018, with updates published in 2019 and 2020. A further update is due to be published in the summer of 2022. It is this update that provides the final list of studies from which this review will draw. The search for this update (EGM 4th edition) was completed in September 2021. Other potential studies will be identified through a call for grey evidence and hand‐searching key journals.

**Selection Criteria:**

Eligible studies will be impact evaluations with designs at levels, 3, 4 and 5 of the Maryland Scientific Methods scale. This therefore includes all studies categorised as either ‘Randomised Controlled Trials’ or ‘nonexperimental designs with a comparison group’ from the studies which form the basis of the Homelessness Effectiveness Studies Evidence and Gap Maps (EGM) created by CHI and the Campbell Collaboration. We are interested in studies that examine the effect of interventions on substance use outcomes. Studies to be excluded are those with designs at levels 1 and 2 of the Maryland Scientific Methods scale, for example, studies without a control or comparison group, ‘before vs. after’ designs (without an untreated comparison group), and cross‐sectional regressions.

**Data Collection and Analysis:**

Descriptive characteristics and statistical information in included studies will be coded and checked by at least two members of the review team. Studies selected for the review will be assessed for confidence in the findings using a critical appraisal tool for determining confidence in primary studies. Standardised effect sizes will be calculated and, if a study does not provide sufficient raw data for the calculation of an effect size, we will attempt to contact the author(s) to obtain this data. We will aim to use random‐effects meta‐analysis and robust‐variance estimation procedures to synthesise effect sizes. If a study includes multiple effects, we will carry out a critical assessment to determine (even if only theoretically) whether the effects are likely to be dependent. Where we suspect dependent effects, we will determine whether we can account for these by robust variance estimation. We will explore the moderating influence of participant and study characteristics, such as gender, race, substances targeted and length of follow‐up. Where effect sizes are converted from a binary to continuous measure (or vice versa), we will undertake a sensitivity analysis to investigate the effect of the inclusion of studies with a converted effect size in the meta‐analysis by running an additional analysis with these studies omitted. We will also assess the sensitivity of results to inclusion of non‐randomised studies and studies classified as low confidence in findings. All analyses will include an assessment of statistical heterogeneity. Finally, we will undertake analysis to assess whether publication bias is likely to be a factor in our findings.

## 1 BACKGROUND

### 1.1 The problem, condition or issue

#### 1.1.1 The significant and increasing scale of homelessness in high income countries

Homelessness is a major social and public health concern (MacKnee & Mervyn, 2002). In recent years, rates of homelessness are reported to have increased in many high income countries, although differences in definitions and measures mean that it is challenging to get an accurate overall picture (OECD, 2020). For example, in the United States, the recent *State of Homelessness in America* report revealed that over half a million people each night are homeless, of whom around 200,000 are experiencing street homelessness (The Council of Economic Advisers, 2019). In Canada, around 35,000 people are homeless each night, with between 250,000 and 300,000 experiencing homelessness a year (Gaetz et al., 2016; Wong et al., 2020). Homelessness continues to rise in most EU countries (FEANTSA, 2017). In the UK, all forms of homelessness have risen since 2008 (O'Leary & Simcock, 2020), and it is estimated that 280,000 people are homeless in England (Shelter, 2021).

Homelessness is a traumatic experience, which can have a devastating effect on those experiencing it. Several studies have highlighted that more visible and extreme forms of homelessness are associated with adverse childhood events, extreme social disadvantage, physical, emotional and sexual abuse, neglect, low self‐esteem, poor physical and mental health, and much lower life expectancy compared to the general population. This group of homeless people experience severe and multiple disadvantages (Bramley et al., 2020) and have complex needs (Dobson, 2019). They are increasingly the focus of policy interest, both here in the UK and elsewhere, and there is a growing recognition that ‘groups experiencing problems such as homelessness, drug and alcohol misuse, poor mental health, and offending behaviours are often populated to a large extent by the same people' (Bramley et al., 2020). They often face a ‘tri‐morbidity' (Cornes et al., 2018); a combination of poor physical health, mental health, and problematic substance use (Cornes et al., 2018; Dobson, 2019; Luchenski et al., 2018; Renedo & Jovchelovitch, 2007), and that longer periods of homelessness are associated with greater severity of these issues (Mayock et al., 2011).

It is increasingly recognised that this group of adults experiencing homelessness face significant barriers accessing services, and often fall through the cracks between different services they need to access (Dobson, 2019). They have repeated, but intermittent, contact with a range of publicly funded services, particularly health (Aldridge et al., 2018), criminal justice (Bramley et al., 2020), and local government (Dobson, 2019). For example, this population is five times more likely to attend accident and emergency, and three times more likely to be admitted to hospital, than their housed peers (Cornes et al., 2018). It is also the case that Covid‐19 presented unique challenges in relation to this population. People experiencing homelessness are more at risk of catching Covid because of generally poor health, and because of the degree of personal contact they might have, especially if living in temporary accommodation or hostels. Public health measures in a number of jurisdictions specifically targeted people experiencing homelessness to reduce their risk of catching or spreading Covid. In the UK, these measures included the provision of temporary accommodation to all individuals experiencing street homelessness (known as *Everybody In*), as well as banning evictions in the private and social rented sectors (Whitehead et al., 2021).

We recognise that homelessness is a complex and multifaceted concept, with differences in how homelessness is understood and experienced, and how these differences are conceptualised and described. We also recognise that there are substantive differences in access to resources and services, and in relation to drivers of homelessness, between high and low income countries. There are also ongoing policy and practice debate around the causes of homelessness, and around interventions aimed at preventing and reducing homelessness. In terms of the causes of homelessness, Suzanne Fitzpatrick states that there is significant debate between a focus on individual‐level risks or causes, and structural or systemic causes (such as labour market conditions, housing supply, and poverty). These foci vary between countries and over time, though increasingly it is recognised that both might have explanatory power (Fitzpatrick, 2012). These debates often influence policy debates around the types of interventions that might address homelessness, and whether these should be focused on increasing housing supply or reducing poverty, or preventing/addressing homelessness at the level of the individual. It is important to note that most of the extant evidence base from high income countries that examines the effectiveness of interventions aimed at reducing or stopping problematic substance use are focused on individual‐level interventions.

#### 1.1.2 The relationship between homelessness and substance use

Substance use is disproportionately high among people who are homeless or vulnerably housed (Aldridge et al., 2018; Magwood et al., 2020). This is a complex relationship; substance use can be the cause and consequence of experiencing homelessness (Peng et al., 2020); indeed, a significant gap in the extant literature is the extent to which substance use and homelessness are both the result of other causes. A large proportion of people experiencing both homelessness and problematic substance use are dependent on the substances they use (Chen et al., 2006; Martijn & Sharpe, 2006; Thompson et al., 2009), largely because of the functional purpose they serve. These include alleviating the stress of life on the streets (Klee & Reid, 1998a, 1998b; Thompson, 2005), helping users to keep warm (Ayerst, 1999), self‐medicating because of physical and mental health problems (Fountain & Howes, 2002; Klee & Reid, 1998a, 1998b), or to block out memories of previous trauma or abuse (Carver et al., 2020; Homeless Link, 2014). Substance dependency presents one of the key challenges in successfully transitioning homelessness individuals into stable housing (Tsemberis, 2011).

Drug overdose is a major cause of death among people experiencing homeless (Bauer et al., 2016), and drug poisoning has contributed to the biggest rise in deaths of people experiencing homelessness in England and Wales since records began. (More than doubled to 726, accounting for more than half of all deaths of people experiencing homelessness [ONS, 2019]). People experiencing homelessness who use drugs are particularly vulnerable to new synthetic drugs, with over 60 drug‐related deaths attributed to synthetic cannabinoids in New Zealand in 2018 (New Zealand Drug Foundation, 2018). In the United States, people experiencing homelessness have higher mortality rates by (synthetic) opioid overdose than national averages (National Health Care for the Homeless Council, 2017).

We recognise that substance use is a controversial topic, in which a variety of approaches and understandings exist relating to evidence in policy and practice. We propose to define ‘substance use’ as any psychoactive compound with the potential to cause health and social problems, including addiction. We exclude tobacco but include other legal substances such as alcohol and prescribed drugs such as benzodiazipines and gabapentinoids. We also include new psychoactive substances such as synthetic cannabinoids and substances such as amphetamine, cannabis, crack cocaine and heroin.

### 1.2 The interventions

#### 1.2.1 Introduction

There are a large number of different interventions that aim to stop, reduce or prevent problematic substance use. These interventions originate in different areas of social policy, including healthcare, criminal justice, education, and housing. We have set out the key interventions to be covered by this review in a typology in Table 1. The focus of this review is to understand whether these interventions are effective and, if so, which are effective and which are not. A second objective is to understand whether interventions that adopt a harm reduction approach are more or less effective than interventions that adopt an abstinence‐based approach.

**Table 1:** Proposed intervention typology

	Abstinence‐based (low tolerance)		Harm reduction based (high tolerance)
Psychosocial interventions	Therapeutic communities	Motivational interviewing	Harm reduction pyschotherapy
	Self‐help (12‐step, AA, NA, Smart Recovery)	Talking therapies	Education regarding safer injecting techniques and not sharing
	Residential rehabilitation	Assertive outreach	Group work (including RAMP)
	Abstinence‐based day programmes	Motivational Enhancement Therapy (MET)	Harm reduction‐based day programmes
	Trauma therapies (including EDMR)	Assertive Community Therapy	
		Intensive Case Management	
		Behavioural Couples Therapy	
Treatment through medication	Detoxification	Agonist pharmacotherapy /blockers	Opioid Substitution Treatment (OST)/Maintenance therapy
			NX provision
			Naloxone
			Rapid prescribing
			Testing for BBVs
			Needle exchanges
Non‐medication intervention	Contingency Management (testing, treatment engagement)		Heroin Assisted Therapy (HAT)
	Recovery Housing		Safe Injecting space/DCRs

#### 1.2.2 Treatment approaches: Harm reduction versus abstinence‐based recovery models

There is ongoing discourse and debate within drug policy and practice around harm reduction and abstinence‐based approaches. Sometimes this debate is framed as an either/or dichotomy (Dennis et al., 2020); other times it is framed as a need to integrate both in a continuum of approaches, ranging from high tolerance harm reduction to low or zero tolerance abstinence‐based approaches. In some cases, such as substitute prescribing of methadone or buprenorphine to heroin users, the same intervention can be viewed as both an abstinence and harm reduction model. The long‐term goal may include reducing the daily amounts used or to stop using the substitute medication completely. However, some abstinence models may require complete abstinence, including from opioid substitution medication.

There is a more substantive empirical literature about the effectiveness of abstinence and use reduction approaches than exists around harm reduction. This reflects the fact that most of the effectiveness literature emanates from North America (and particularly the United States), where abstinence has historically played a more central role in substance misuse treatment compared to Europe, the UK and elsewhere (Bujarski et al., 2013). It is also reflective of abstinence being a core criterion of recovery (Von Greiff et al., 2020), particularly for medical centred interventions (Demartini et al., 2014).

It is also important to differentiate between abstinence as a goal or anticipated outcome of a substance use intervention, and abstinence as a conditional requirement for access to non‐substance misuse treatment services such as housing and mental health services. It is also important to differentiate between abstinence that is required of a person experiencing homelessness, and abstinence as a goal that an individual sets themselves.

#### 1.2.3 How the interventions might work

Abstinence‐based and harm reduction based interventions likely draw on different causal assumptions about how individuals might address or manage their substance misuse, even where they might have similar goals or outcomes in relation to abstaining from substance use. Broadly speaking, the theoretical underpinning of harm reduction approaches is more developed in the literature than in relation to abstinence‐based interventions. Indeed, in a paper about the ‘active ingredients' of effective substance misuse treatments, Rudolf Moos (Moos, 2007) identifies four potential causal explanations underpinning different treatments, but concludes that little is known about how these different social processes generate reductions in substance misuse. These theoretical assumptions appear not to be linked to theories about why individuals engage in addictive behaviours. Drawing on this limited theoretical work, we identify two possible ‘theories of change' underpinning abstinence and harm reduction approaches to substance misuse treatment for homeless adults, namely social control (which underpins abstinence‐based interventions) and self control (which underpins harm reduction based interventions) We anticipate that these theories will be further developed through the systematic review and policy guidance work; at this stage, we summarise these two theories of change in the next two sections.

#### 1.2.4 Social control

Abstinence programmes such as 12 step programmes draw on social control theory (Moos, 2007). According to social control theory, strong social network bonds and structure can motivate individuals to engage in ‘responsible’ behaviour and refrain from substance use and other deviant pursuits. In the absence of such bonds, individuals are less likely to adhere to conventional standards and tend to engage in undesirable behaviour, such as the misuse of alcohol and drugs (Moos, 2007). Abstinence‐based interventions are forms of overt social control; organised mechanisms through which power is used to manipulate, change or prevent behaviours that are seen as problematic (Cohen, 1985; Johnsen et al., 2018). The use of terms such as criminalise, punish, discipline to describe such approaches is indicative of this use of power in social control; it is a top down use of *power over* people experiencing homelessness who misuse drugs and alcohol. Abstinence removes control and decision from people experiencing homelessness (Woodhall‐Melnik & Dunn, 2016). People experiencing homelessness are passive ‘objects’ who require this paternalism (Whireford, 2013).

Examples of interventions drawing on social control theory include therapeutic communities, detoxification, and 12 step programmes.

Therapeutic communities are a participatory, group‐based, and often residential approach to addressing substance use and mental ill health. The community is seen to be the key agent of change in this intervention, where community involves both those being treated, and therapists (Smith et al., 2006). Abstinence is a core requirement of participation in therapeutic communities. Detoxification interventions involve the use of pharmacological treatments to address withdrawal symptoms associated with abstaining from problematic substance use. Detoxification can be used in a number of different settings, including clinical and community approaches. Detoxification is part of an overall treatment plan that aims to achieve and maintain abstinence from substance use. Like therapeutic communities, 12 step programmes are participatory, group‐based interventions. These programmes use peer support and a recognition that the individual cannot control their addiction to achieve and maintain abstinence.

#### 1.2.5 Self‐control

Harm reduction approaches draw on a presumption of individual autonomy (Hawk et al., 2017); that individuals are best placed to know their own interests. Harm reduction‐based services and often assume that ensuring people experiencing homelessness should have agency and choice about their housing and other support services (Whireford, 2013). Bowpitt and colleagues (Bowpitt et al., 2014) for example talk about day centres providing ‘supportive enablement’, that provide people experiencing homelessness with the facilities to ‘negotiate their own cases with other agencies’. Much of the literature focuses on the harms of problematic substance use and on specific harm reduction strategies, rather than on an overall harm reduction philosophy (Hawk et al., 2017). Harm reduction approaches are rooted in the importance of an individual's agency in their substance use treatment. This includes (a) accepting that substance use is an individual choice; (b) not coercing or requiring abstinence or change, or making access to wider services contingent on abstinence or change; and (c) coproduction [a commitment to ‘the meaningful involvement of people who use drugs in designing, implementing and evaluating programmes and policies that serve them is central to harm reduction’. (HRI, 2022)]. In contrast to the top down use of *power over* people experiencing homelessness that is core to abstinence conditionality, non‐interventionist approaches aim to use *power to* enable individuals to make choices and decisions for themselves. Examples of interventions that draw on self‐control theory include opioid substitution therapy, safe injecting spaces, and harm reduction psychotherapy.

Opioid substitution therapy is an intervention that includes pharmacological and psychosocial treatment. This is a harm reduction intervention, which aims to reduce the harms associated with opiod use, but not to stop substance use altogether. This type of therapy is seen as developing self‐efficacy and autonomy (Ayres et al., 2014; Skjaervo et al., 2021) and, as such, can be associated with self‐control theory. Safe injecting spaces are an intervention that seek to reduce the risks associated with sharing syringes, injecting in non‐sterile or unsafe venues. The intervention involves the use of clean syringes under medical supervision. They are often part of a package of measures aimed at improving self‐care and self‐efficacy. Harm reduction psychotherapy emerged in the late 1990s, and is rooted in an assumption that people engaging in problematic substance use have agency and autonomy, and that service user choice is an important goal of treatment (Milet et al., 2021). This intervention is associated with improved self‐efficacy (Pawa & Areesantichai, 2016) and can therefore be seen as being underpinned by self‐control theory.

We anticipate that these two theories will be further developed and critically evaluated through the systematic review. There are three key issues that need to be resolved with respect to these theories of change.

First, it is possible to use either self‐control or social control frameworks to explain how some individual interventions might work, or to incorporate both in explaining individual interventions. Twelve Step programmes are an example of this. There are several elements of 12 Step programmes that reflect social control approaches; that the individual engaging in problematic substance use has no control over their use, and group reinforcement to achieve and maintain abstinence, as well as references to higher powers. But 12 Step programmes can also be framed as self‐control, as a self‐help intervention that relies on peer support. Second, a significant gap in the relevant empirical literature is the lack of explanation of how the intervention might work. Few explain why expected outcomes should flow from the intervention, or how the intervention will engender change in problematic substance use and related behaviours. Finally, there are a small number of interventions that employ either harm reduction or abstinence only approaches, generally based on the jurisdiction within which the intervention is implemented. This would suggest that debates in the literature about harm reduction versus abstinence dichotomy might not be material to intervention effectiveness.

### 1.3 Why it is important to do this review

#### 1.3.1 Policy relevance

Homelessness is a significant and growing policy issue in a number of countries around the world. Over the past decade or so, rates of all kinds of homelessness have increased in many high income countries. It is increasingly recognised that homelessness has a devastating effect on those experiencing it, on the wider community, and is costly to the public purse. There are ongoing debate around which interventions are most effective in preventing and reducing homelessness, particularly in relation to people experiencing severe and multiple disadvantage homelessness. This is a particularly acute form of homelessness, where people experiencing homelessness also face issues around problematic substance use, offending behaviour, and/or mental health issues. People experiencing this form of homelessness typically make extensive and expensive demands on public services, notably in health (Aldridge et al., 2018) and criminal justice (particularly as people experiencing street homelessness are considerably more likely than the general population to be victims of crime, (O'Leary, 2004). There is also evidence to suggest that they face issues accessing services (Cornes et al., 2018), and that evidence of the effectiveness of interventions is somewhat mixed (Luchenski et al., 2018).

It is also a significant issue in substance use policy debates, around different types of treatments and interventions targeting problematic substance use. This policy debate is complicated by three issues with the underlying effectiveness studies. First, there is a significantly greater body of effectiveness studies around abstinence‐based interventions compared to harm reduction interventions, which can make comparisons difficult; second, because some interventions are not specifically harm reduction or abstinence‐based; and, third, because there is mixed evidence of effectiveness, with individual studies suggesting that some interventions using both harm reduction and abstinence‐based approaches are effective and others are not.

There is also a significant gap in the extant effectiveness evidence in terms of the voice of people with lived experience of homelessness, and largely treats people with lived experience as passive research participants. This proposed review aims to elevate the voice people with lived experience in two ways. First, there will be an experts by experience review process that will run alongside the technical peer review process. This will enable the review team to gain views on relevance and appropriateness of the outcomes in the underlying studies. Second, the team proposes to work with a panel of people with lived experience to coproduce the discussion, recommendations, and conclusions of the published review.

Policy makers interested in using the evidence to determine whether and what types of interventions are most effective therefore face considerable challenges in navigating and interpreting the extant effectiveness evidence base. This systematic review aims to provide a single synthesis of the evidence base to aid policy makers in their decisions.

#### 1.3.2 Previous reviews

There are two existing systematic reviews relevant to problematic substance use by people experiencing homelessness. The review proposed here will complement these two reviews.

Magwood et al. (2020) completed a review of reviews of the effectiveness of specific harm reduction interventions, including two pharmacological harm reduction interventions, safe consumption rooms, and managed alcohol programmes. The reviewers identify that there has been no previous evidence synthesis specific to people experiencing homelessness. They also report that initial searches did not find any relevant studies about homelessness, and that they expanded their search to include the general population. Our proposed study complements, but does not replicate, the review by Olivia Magwood and colleagues. Our review seeks to examine the effectiveness of harm reduction and abstinence only interventions, and examining differences in effects between these types of interventions, whereas the Magwood study focuses on the effectiveness of specific harm reduction interventions. Our review thus addresses a different research question. Our review is focused on interventions specifically targeted at homeless populations, whereas the Magwood review of reviews considered the general population. While, as Magwood and colleagues make clear, the studies included in their review included a large number of people experiencing homelessness (and indeed the underlying evidence in relation to managed alcohol programmes almost completed related to this population), the review proposed here will be specific to this population.

The second relevant review is that by Aliza Moledina and colleagues, published in June 2021 (Moledina et al., 2021). This review examined a range of different interventions aimed at people experiencing homelessness, including housing, income assistance, case management, mental ill health, and problematic substance use interventions. The Moledina et al. review is focused on people experiencing homelessness, whereas the Magwood et al review outlined above is broader in terms of target population. Although the Moledina et al review covers a number of substance use interventions, as with the Magwood review, it focuses only on a small number of harm reduction interventions. As such, it also addresses a different research question to that which underpins the review outlined in this protocol.

There are existing reviews and protocols (e.g., Adams‐Guppy & Guppy, 2016; Carver et al., 2020; Vijayaraghavan et al., 2020) which focus on interventions designed to reduce substance abuse in homeless populations, but these have tended to focus on specific substances (e.g., alcohol, tobacco) or have included substance abuse outcomes within the broader remit of investigating the effect of interventions on health. A recently published systematic review on accommodation‐based services for people experiencing homelessness by Ciara Keenan and colleagues (Keenan et al., 2021) examined a number of outcomes related to the provision of accommodation and related support services, including health and criminal justice outcomes. The Keenan et al. review did not specifically look at substance use interventions or outcomes.

## 2 OBJECTIVES

The review will aim to answer the following questions:

1. How effective are interventions designed to reduce substance use in adults who are experiencing homelessness compared to treatment‐as‐usual?
2. What is the effect of abstinence‐based interventions on substance use outcomes in adults who are experiencing homelessness?
3. What is the effect of harm‐based interventions on substance use outcomes in adults who are experiencing homelessness?
4. Are abstinence‐based interventions more or less effective than harm reduction‐based interventions?
5. What is the effect of individual interventions designed to reduce substance use in adults who are experiencing homelessness, compared to treatment as usual and each other?
6. How do participant and study characteristics moderate the effect of interventions designed to reduce substance use in adults who are experiencing homelessness? Specifically:
  a. For which substances are interventions most effective?
  b. For whom do the interventions work best?
  c. Over what period of time are interventions most effective?
  d. How does the length of follow up period moderate effectiveness?

## 3 METHODS

### 3.1 Criteria for considering studies for this review

#### 3.1.1 Types of studies

Eligible studies will be impact evaluations with designs at levels, 3, 4 and 5 of the Maryland Scientific Methods scale^1^:

- Level 3. Comparison of outcomes in treated group after an intervention, with outcomes in the treated group before the intervention, and a comparison group used to provide a counterfactual (e.g., difference in difference) … techniques such as regression and (propensity score matching may be used to adjust for difference between treated and untreated groups.
- Level 4. Quasi‐randomness in treatment is exploited, so that it can be credibly held that treatment and control groups differ only in their exposure to the random allocation of treatment. This often entails the use of an instrument or discontinuity in treatment, the suitability of which should be adequately demonstrated and defended.
- Level 5. Reserved for research designs that involve explicit randomisation into treatment and control groups, with Randomised Control Trials (RCTs) providing the definitive example. Extensive evidence provided on comparability of treatment and control groups, showing no significant differences in terms of levels or trends.

This therefore includes all studies categorised as either ‘Randomised Controlled Trials’ or ‘nonexperimental designs with a comparison group’ from the studies which form the basis of the Homelessness Effectiveness Studies Evidence and Gap Maps (EGM) created by CHI and the Campbell Collaboration (Singh & White, 2022 (expected); White, 2018; White & Narayanan, 2021; White et al., 2019).

Studies to be excluded are those with designs at levels 1 and 2 of the Maryland Scientific Methods scale, for example:

- studies without a control or comparison group
- ‘before vs. after’ designs (without an untreated comparison group)
- Cross‐sectional regressions

As the review will therefore include randomised and non‐randomised studies we will undertake a sensitivity analysis to investigate the effect of the inclusion of non‐randomised studies in the meta‐analysis.

#### 3.1.2 Types of participants

There are a number of definitions of homelessness available, reflecting differences between countries and over time. There are also different forms of homelessness, considering the length of time someone has been experiencing homelessness, distinctions between living on the street or in their vehicles, or having a temporary place to stay.

We propose to draw on the definition of homelessness used in a recently published Campbell Collaboration protocol. This definition is:

Homelessness is defined as those individuals who are sleeping rough (sometimes defined as street homeless), those in temporary accommodation (such as shelters and hostels), those in insecure accommodation (such as those facing eviction or in abusive or unsafe environments) and those in inadequate accommodation (environments which are unhygienic and/or overcrowded).

(Keenan et al., 2020)

Our focus is on adults experiencing (men and women aged 18 years and over), undertaken in any high‐income country and published in English. Studies of families or children will be excluded from the review. In many countries (particularly the UK), there are different legal frameworks that apply to homeless families and children, and thereby access to different types of services, and different outcomes expected. The review will focus on homelessness in high income countries, thereby recognising the substantive differences between high‐ and low‐income countries in terms of access to resources and services, and drivers of homelessness (Magwood et al., 2020).

#### 3.1.3 Types of interventions

The review will synthesise findings about the impact of a range of interventions aimed at stopping or reducing substance use (including interventions which also aim to reduce associated harms). In developing this protocol, and drawing on the extant effectiveness literature, the review team has created a typology of interventions. This typology is set out in Table 1. The typology was discussed and validated with an expert panel of academics, policy makers, experts by experience, and practitioners involved in problematic substance use treatments targeted at people experiencing homelessness held in November 2020.

Interventions listed in the left‐hand column are abstinence‐based; interventions in the right‐hand column are based on harm reduction approaches:

i) Abstinence‐based—Interventions that require participants to abstain from substance use, or whose goal is to achieve abstinence from substance use.
ii) Harm reduction—Interventions that seek to reduce the harm caused by substance use but which do not require abstinence.

Interventions listed in the centre column are often practiced using both abstinence‐based and harm reduction approaches. These interventions are relevant to addressing the first objective of this review (how effective are interventions designed to reduce substance use in adults who are experiencing homelessness).

Interventions aimed at reducing problematic substance abuse can also be categorised descriptively by type. The review team has identified three such categorisations, namely psychosocial, treatment through medication (pharmacological), and nonmedical interventions. These descriptive categories are not material to the proposed analysis or to addressing the review objectives.

Data extracted from studies included in the review will include details of the comparison made with the treated group in each individual study (e.g., no intervention, treatment as usual, wait‐list, other treatment). We do not expect that the majority of included studies will make a direct comparison between different types of intervention.

#### 3.1.4 Types of comparison

We will consider studies for inclusion in the review that used the following types of control or comparison group:

- Treatment‐as‐Usual (TAU): We will include studies that compare outcomes of people offered an intervention in our typology to a group of people experiencing the TAU offer. We anticipate that most studies included in the review will fall into this category. We will capture whether the TAU offer appears to be primarily abstinence‐based or harm reduction‐based in our data extraction tool, to the extent that this information is presented in the original studies.
- Another intervention: We will include studies that compare outcomes of people receiving a relevant intervention in our typology to a group of people receiving another new intervention. Again, we will capture whether the comparison group intervention appears to be an abstinence‐based approach or a harm reduction‐based approach in our data extraction tool, to the extent that this information is presented in the original studies.
- No treatment: In theory, we will include studies that compare outcomes of people receiving a relevant intervention in our typology to a group of people receiving no treatment offer. However, we do not anticipate that we will identify any studies where there is no existing substance use treatment offer, including for people experiencing homelessness.
- We will include studies that used a wait‐list design or those that did not offer the relevant intervention to the control group at the end of the study.

We will use studies with multiple arms comparing abstinence vs harm vs a control group or those that make a direct comparison between abstinence‐based approaches and harm reduction approaches to answer our fourth research question, that is, are abstinence‐based interventions more or less effective than harm reduction‐based interventions.

#### 3.1.5 Types of outcome measure

The review will investigate substance use outcomes, primarily identifying studies by outcome from studies included in the Homelessness Effectiveness Studies Evidence and Gap Map (EGM) created by CHI and the Campbell Collaboration (Singh & White, 2022 (expected); White, 2018; White & Narayanan, 2021; White et al., 2019). We will also undertake an additional review of all studies contained in the EGM to search for more studies with a substance use outcome.

Substance use is measured in a number of ways by primary studies, for example:

- Number of days per month substances are used
- Self‐reported measures of drug‐related problems
- Drug use reduction programme participation
- Drug testing

We expect a range of continuous and binary outcomes to feature in the reviewed studies, and we will convert these into the same metric (e.g., Hedges' *g*) for meta‐analysis (Borenstein et al., 2009). Where effect sizes are converted from a binary to continuous measure (or vice versa, depending on our ultimate choice of effect size), we will undertake a sensitivity analysis to investigate the effect of the inclusion of studies with a converted effect size in the meta‐analysis.

### 3.2 Search methods for identification of studies

The primary source of studies for potential inclusion in this review is the Homelessness Effectiveness Studies Evidence and Gaps Maps (EGM). The first of these was published in 2018 (White, 2018), with updates published in 2019 (White et al., 2019) and 2020 (White & Narayanan, 2021). A further update has been published in the summer of 2022 (Singh & White, 2022). It is this update that provides the final list of studies from which this review will draw. The search for this update (EGM 4th edition) was completed in September 2021. Other potential studies will be identified through a call for grey evidence and hand‐searching key journals.

#### 3.2.1 The homelessness effectiveness studies EGM

The process for identifying and searching for the studies included in the EGMs is described by White and Narayanan (2021). For further details see Appendix A. Initial searches were carried out in 2018, and 260 studies were included in the first iteration of the EGM. The most recent search (upon which this review is based) was completed in September 2021 and includes 562 studies in total. Each new edition of the EGM involves a new search being undertaken, with several studies added in each iteration.

Sources of studies for potential inclusion in the EGM included (White & Narayanan, 2021):

1. Approx. 140 RCTs identified by Munthe‐Kaas et al. (2016, cited by White & Narayanan, 2021)
2. 30 systematic reviews identified during the scoping of the EGM
3. A full database search to identify primary studies and systematic reviews
4. An additional website search for grey literature
5. Unpacking systematic reviews in i‐iii above to identify additional primary studies
6. Consultation with subject matter experts
7. Additional studies received in response to dissemination of early iterations of the EGM

The reviewers will begin to shortlist studies for this review by identifying primary studies and systematic reviews from the EGM (2020 and 2022 editions) (by filtering using the previously coded classifications) which appear to meet the inclusion criteria for this review. Systematic reviews from the EGM list will be unpacked, and their constituent studies added to the shortlist, along with studies identified through the call for grey evidence and handsearching key journals (see below).

#### 3.2.2 Searching other resources

In November 2020 the review team issued a *call for grey evidence* (with a deadline of 8th January 2021) which was disseminated through the review team's and review funder's social media channels, inviting people with lived experience, researchers, commissioners, service providers and wider stakeholders to submit relevant grey evidence for consideration in the review. Specifically, the call was for evidence that is:

- empirical, based on research that:
  ∘ elevates the voice of people with experience of homelessness;
  ∘ measures the impact of interventions (before and after, quasi‐experimental, randomised controlled trial);
  ∘ identifies the barriers to, and facilitators of, successful implementation of interventions;
- is about interventions aimed at reducing or stopping problematic substance misuse, using harm reduction or abstinence‐based approaches;
- is not published in a book or academic journal; and
- is specific to the UK, or England, Northern Ireland, Scotland or Wales.

We received eight studies in response to the call, none of which were impact evaluations or systematic reviews, and hence will not be eligible for inclusion in the review.

The reviewers will also *hand search key journals*, using similar search terms and date ranges as White et al. 2020. While some may have already been searched as part of the evidence and gap map (White & Narayanan, 2021), this targeted journal search and more substance use and treatment focused search will further ensure the capture of all existing literature and evidence. The hand searched journals will include:

- *Drug Alcohol Dependency*
- *Psychiatric Services Journal*
- *American Journal of Public Health*
- *BMJ*
- *Journal of Substance Abuse Treatment*
- *European Journal of Homelessness*
- *Housing Studies*
- *Social Policy and Administration*
- *Addiction Research and Theory*
- *Drugs Education Prevention and Policy*
- *International Journal of Drug Policy*
- *Addiction*

### 3.3 Data collection and analysis

#### 3.3.1 Description of methods used in primary research

Primary research will be based on designs at levels 3, 4 and 5 of the Maryland Scientific Methods scale, including experimental (randomised) and quasi‐experimental studies. Such studies will measure the effectiveness of an intervention designed to reduce problematic substance use against another intervention or a control group (e.g., no intervention, treatment as usual, wait‐list) or another intervention.

#### 3.3.2 Screening

Studies will be selected from the EGM (3rd and 4th editions) by initially filtering using the previously coded classifications. A subsequent manual search of the list will be employed as a second stage for quality assurance. Further studies will be added to the shortlist from four sources:

1. unpacking systematic reviews contained in the EGM list
2. the call for grey evidence
3. handsearching key journals
4. electronic search of key journals (string used for this search is set out in the appendix)

The shortlist of studies identified from the sources described in 3.2 will be screened in two stages; (i) title/abstract, (ii) full‐text) using the inclusion/exclusion criteria defined in Section 3.1. All screening will be undertaken by two reviewers, and any disagreements will be escalated to two subject matter experts on the review team. Twenty five percent (25%) of final screening decisions will be sampled by a third reviewer. Final decisions about inclusion will be made by four members of the review team.

#### 3.3.3 Data extraction and management

Data will be extracted from eligible studies by two reviewers, to include details of the study, quantitative data required for meta‐analysis, and confidence in the study's findings (using the Campbell Collaboration's critical appraisal tool for primary studies—White & Narayanan, 2021). Coding disagreements will be discussed and if necessary passed to the lead reviewer for resolution. We will extract data for the following, where it is not already present in the EGM spreadsheet:

- Publication details (e.g., authors, year, source, study location)
- Intervention details, including basis (e.g., abstinence‐based or harm reduction‐based) and typology classification
- Substance and substance classification (e.g., alcohol, cannabis and synthetic cannabinoids, opiates and opioids, stimulants, CNS depressants, hallucinogens)
- Participant details, including classification (e.g., age, gender)
- Study design
- Comparison (e.g., other intervention, treatment as usual, waitlist control)
- Outcome description, definition and measurement (including measurement duration)
- Sample sizes of treatment and control groups
- Data to calculate odds ratios, rate ratios or standardised mean difference (SMD)
- Confidence assessment

#### 3.3.4 Assessment for confidence in the included studies

Studies selected for this review that are included in the EGM have been assessed for confidence of findings using two separate critical appraisal tools: one for primary studies and one for systematic reviews. Details of these tools and the assessment process are set out in White et al. (2019); and the tool itself can be found in appendix C. The critical appraisal tool used for primary studies is one developed by the Campbell Collaboration for use with maps and reviews. The tool for primary studies has seven items which relate to (1) study design, (2) blinding, (3) power calculations, (4) attrition, (5) description of the intervention, (6) outcome definition and (7) baseline balance. Each of these seven items is rated as implying high, medium or low confidence in study findings. Overall quality is assessed using the ‘weakest link in the chain’ principle, so that confidence in study findings can only be as high as the lowest rating given to any of the critical items.

The assessments undertaken by the Campbell Collaboration form the basis of our quality assessment and we do not propose to undertake further assessment on these studies. Studies that are identified through the additional searches outlined above, or any studies unpacked from systematic reviews, which are not listed in the EGM and therefore have not been assessed, will be subjected to assessment of confidence using this tool. Classifications will be undertaken by one researcher and judgements (high/medium/low confidence) will be verified by a second researcher who will sample check 25%. Sensitivity analyses will be undertaken to determine the effect of excluding studies classified as low confidence, effectively by running an additional analysis with these studies omitted (see section below).

#### 3.3.5 Criteria for determination of independent findings

Dependent effects can occur when a study reports results for multiple measures of the same outcome construct for the same sample, the same outcome measure at multiple time points, when a study has multiple treatment arms compared to a common control group, or multiple studies evaluate the same programme and report on the same outcome. This is problematic as estimating an average effect using standard meta‐analytic models rely on the statistical assumption of independence of each included effect size (Gleser & Olkin, 2009).

Once we have identified our pool of included studies, we will map the programmes being evaluated, the outcome measures used in each study and the follow‐up time(s) to identify possible dependent effects. We will implement the following strategies to address dependent effects, drawing on Pigott and Polanin (2020), with an aim to balance capturing as much relevant information from each study as possible with the limited timeframe for the review:

- Multiple follow‐up points: If a study reports results for the same outcome at multiple follow‐up points, we will extract data for a maximum of two time points that also meet the inclusion criteria of following up at 36 months or less and calculate standardised effect sizes for both. We will extract a short‐term follow‐up (the closest to the 6‐month follow‐up point) and a longer‐term follow‐up point. We will extract the closest result to a 6‐month follow‐up as this is a key point of interest for policymakers for this sort of intervention. We will map the longer term follow‐ups across all studies in the review and then extract the longer term follow‐up point that is most common across each study. We will attempt to explore variation in impacts by time of follow‐up (i.e., research question 8) using meta‐regression or sub‐group analysis.
- Multiple measures of the same construct: If a study reports results for multiple outcomes measuring the same or similar construct, we will extract one measure within two groups of outcome measures from each study: one self‐reported measure of substance use and one objective substance use measure (e.g., results of a drug test), using the most commonly used measures across the selected studies. Within these two categories of outcomes, if there are multiple self‐report measures or multiple objective measures in a single study, we will decide on which to extract based on which is most common across the review. This is with the exception of measures broken down by type of substance. If a study presents a general measure of substance use, we extract that over individual measures of specific substances that may also have been used. However, if a study only presents multiple measures of substance use broken down by type of substance, we will extract all. We will include in the same meta‐analysis using robust variance estimation (as described above) and explore variation by type of outcome measure.
- Multiple studies on the same programme: If different studies report on the same programme but use different samples (e.g., from different regions), we will include all in the review and include both in the same meta‐analysis, treating them as independent samples. This is provided that the effect sizes were measured relative to a different control group. If multiple studies report on the same outcome(s) with overlapping samples, we will choose the study with the larger sample size for inclusion in the review.
- Multiple treatment arms compared to a common comparison group: if a study reports results for multiple treatment arms and all interventions meet our inclusion criteria, we will extract data for all arms and include in the same meta‐analysis using robust variance estimation (as described above).
- Multiple specifications: If a study reports multiple estimates using different specifications for the same outcome, we will choose the one that the authors present as their primary estimate. We will prioritise the Intention To Treat (ITT) estimate from RCTs where possible as a more conservative and realistic estimate of programme impact.
- Multiple papers on the same study: if we identify multiple reports on the same study (e.g., a journal article and a working paper), we will include the most recent version.

#### 3.3.6 Measures of treatment effect

Effect sizes will be calculated using the esc package in R (effect size computation for meta analysis) (Ludecke, 2019) and Wilson's Practical Meta‐Analysis Effect Size Calculator (Wilson, 2001) If a study does not provide sufficient raw data for the calculation of an effect size we will attempt to contact the author(s) to obtain this data. Where effect sizes are converted from a binary to continuous measure (or vice versa), we will undertake a sensitivity analysis to investigate the effect of the inclusion of studies with a converted effect size in the meta‐analysis by running an additional analysis with these studies omitted (see section on sensitivity analysis). If a study includes multiple effects, we will carry out a critical assessment to determine (even if only theoretically) whether the effects are likely to be dependent.

Where we suspect dependent effects we will determine whether we can account for these by robust variance estimation using the clubSandwich package in R for use of the cluster‐robust variance estimators with small‐sample corrections (Pustejovsky & Tipton, 2021). We will also as far as possible extract consistent measurements across studies, as described above. We will also assess for unit of analysis errors and based on the number of studies which feature such errors we will make a decision about whether to exclude these studies from any meta‐analysis. However, we anticipate that most studies will randomise and analyse data at the individual level and therefore unit of analysis errors will not be an issue for the review.

#### 3.3.7 Data synthesis

The review team undertook early piloting work with studies from the EGM likely to be included in the review. It was noted by the review team that the study interventions and comparison groups were heterogeneous and complex. Given the requirement to synthesise studies with many working components, network meta‐analysis was investigated as it would allow for the possibility of analysing interventions together to determine the contribution of their specific components. However the piloting work found that the interventions formed a number of networks which would make network meta‐analysis unfeasible. The review team therefore decided to proceed with pairwise meta‐analysis as a preferred option for data synthesis.

All analyses will be undertaken using the statistical programming language R, principally using the metafor package (Viechtbauer, 2010), and the clubSandwich package for use of the cluster‐robust variance estimators with small‐sample corrections (Pustejovsky & Tipton, 2021), given that many of the included studies will include multiple relevant effect sizes. Random effects models and restricted maximum‐likelihood estimation will be used to estimate the total amount of heterogeneity. Random effects models are appropriate when the constituent studies differ in terms of mixes of participants and interventions (Borenstein et al., 2009). All analyses will include an assessment of statistical heterogeneity, that is, variability in intervention effects, described in more detail below. In meta‐analyses of social interventions, we expect statistical heterogeneity to be substantial and driven by variability in the underlying studies, which are likely to represent a range of interventions delivered to participant groups with different characteristics, in different locations and at different times.

The analysis approach to address each research question is summarised in Table 2 and described in more detail below. Where meta‐regression is proposed to explore the influence of moderating variables such as gender, these results will be presented as exploratory and with appropriate caution, given that these relationships are observational in nature and likely based on a small number of effects. Before undertaking any of the analysis, we will examine how the moderators of interest relate to one another, for example whether a particular substance is more commonly targeted by a particular type of intervention or whether a particular intervention is typically delivered over a longer period.

**Table 2:** Analysis approach for each research question

Research question	Analytical approach
1. How effective are interventions designed to reduce substance use in adults who are experiencing homelessness compared to treatment‐as‐usual?	Meta‐analysis with robust variance estimation, of all studies included in the review that compare a relevant substance use intervention to a treatment as usual condition.
2. What is the effect of abstinence‐based interventions on substance use outcomes in adults who are experiencing homelessness?	Meta‐analysis with robust variance estimation, including all studies in the review that include a treatment as usual comparison group and with intervention approach (abstinence or harm reduction) included as a moderating variable. Depending on the number of included studies and effect size, we will also include the other moderators of interest as independent variables in the analysis: namely, type of substance, gender, race and length of follow‐up.
3. What is the effect of harm‐reduction interventions on substance use outcomes in adults who are experiencing homelessness?	
4. Are abstinence‐based interventions more or less effective than harm reduction‐based interventions?	Meta‐analysis with robust variance estimation of the subset of studies that make a direct comparison between abstinence based and harm reduction approaches.
5. What is the effect of individual interventions designed to reduce substance use in adults who are experiencing homelessness, compared to treatment as usual and each other?	Meta‐analysis with robust variance estimation by intervention type (e.g., Therapeutic Communities, Assertive Outreach, etc.), broken down by whether the intervention is compared to a treatment as usual comparison group OR another intervention type. If there are insufficient effect sizes to undertake meta‐analysis for a particular intervention type, we will present results narratively.
6. How do participant and study characteristics moderate the effect of interventions designed to reduce substance use in adults who are experiencing homelessness? Specifically:	Meta‐analysis with robust variance estimation of all studies in the review that include a treatment as usual comparison group, including substance, participant characteristics (gender, race) and length of intervention/follow‐up as moderating variables. As described above, we may also include intervention approach (abstinence‐based or harm reduction) as a moderating variable. If it is not possible to include these in a single analysis, because of insufficient number of effect sizes, we will explore the use of single variable meta‐regression, to be interpreted with appropriate caution.
For which substances are interventions most effective?For whom do the interventions work best?Over what period of time are interventions most effective?How does the length of follow up period moderate effectiveness?	

#### 3.3.8 Review question 1

To answer research question 1, we will undertake a meta‐analysis of all included studies for which we are able to calculate a comparable standardised effect size (i.e., Hedge's *g* or odds ratios, depending on the most common types of outcomes reported in the original studies), using the procedures described above. This analysis will combine studies evaluating a range of substance use interventions across our typology and will produce an average effect of interventions designed to reduce substance use in adults experiencing homelessness on substance use outcomes compared to Treatment‐As‐Usual (TAU).

#### 3.3.9 Review questions 2 and 3

To answer research questions 2 and 3, we will categorise studies by whether they evaluate an abstinence‐based approach or harm reduction‐based approach, defined above. We will then aim to undertake a meta‐regression at the review level where intervention approach (harm‐reduction approach or abstinence‐based approach) is included as a moderating variable. We may exclude a small number of studies from this analysis where the approach is not clearly defined as either harm reduction‐based or abstinence‐based. If there are sufficient number of studies/effect sizes included in the review and sufficient variation, we will include the other moderators of interest as variables in the same meta‐regression (see research question 6): namely, type of substance, gender, race, and length of follow‐up. Otherwise, we will attempt single characteristic meta‐regression.

#### 3.3.10 Research question 4

To answer research question 4, we will analyse only the sub‐set of studies that allow us to make direct comparisons between abstinence‐based approaches or harm reduction‐based approaches, described above in section on Types of Comparison. We do not anticipate that there will be many of these studies; however, if possible, we will undertake a meta‐analysis, using the procedures described above. If this is not possible because there are too few studies, we will describe the results of these studies narratively.

#### 3.3.11 Research question 5

To answer research question 5, we will categorise studies by the type of intervention(s) they evaluate, using the typology in Table 1, for example, Therapeutic Communities, Assertive Outreach, etc. We will then aim to undertake separate meta‐analyses of studies categorised into each intervention area, first for studies that compare to either a TAU condition and then for studies that compare to do another intervention. We will take this approach as we expect to find a small number of studies within each category in the typology. Depending on the number of studies and effect sizes included for each intervention type, we may present the results of individual studies narratively.

#### 3.3.12 Research question 6

To answer research question 6, we will code information about the participants targeted in the study, including gender and race, substances targeted, and the length of intervention and the length of follow‐up. If there are sufficient number of studies/effect sizes in the review and sufficient variation in the moderators of interest, we will include all of these variables as moderators in a single meta‐regression at the review level, that is, including all studies for which we are able to calculate a comparable standardised effect size. If there are insufficient number of effect sizes included in the review to undertake meta‐regression, we will attempt single characteristic meta‐regression.

#### 3.3.13 Dealing with missing data

Missing data will be sought as described above, that is, by attempting to contact study author(s) to obtain it. If we are unable to do this then the study will potentially be excluded from analyses, depending on the type of data which is missing.

#### 3.3.14 Assessment of heterogeneity

We will report the effect size and confidence interval for each individual study, and the total amount of heterogeneity (*I* ^2^) for each analysis. This will describe ‘the percentage of variation across studies that is due to heterogeneity rather than chance’.^2^ We will also present *τ* ^2^ for each meta‐analysis, the estimate for the true variance in effect size among the set of included studies.

#### 3.3.15 Assessment of publication biases

As our search strategy includes grey literature, this should help to mitigate any publication bias which might be observed if we were to only include published studies (as published studies are likely to report larger than average effects; Borenstein et al., 2011). We will however undertake additional analysis to assess whether publication bias is likely to be a factor in our findings. This will include a funnel plot to determine whether the summary effects of the meta‐analysis are subject to publication bias, and if this appears to be the case, further tests (e.g., Duval and Tweedie' Trim and Fill) to determine a “best estimate of the unbiased effect size” (Borenstein et al., 2009, p. 286).

#### 3.3.16 Moderator analysis and investigation of heterogeneity

A key goal of meta‐analysis is to identify and analyse heterogeneity in studies included in a review, by exploring whether characteristics of the included studies are associated with variation in effect size. We will therefore carry out moderator analysis as described above, primarily through meta‐regression, to explore observed heterogeneity between the included studies if there are sufficient effect sizes and studies to do so. These analyses will be based on the following categorical moderating variables, assuming sufficient data of the required quality can be extracted:

- Substance classification (e.g., alcohol, cannabis and synthetic cannabinoids, opiates and opioids, stimulants, CNS depressants, hallucinogens)
- Demographics (e.g., gender, race)
- Study location (USA, non‐USA)
- Measurement duration

Depending on the number of effect sizes and number of included studies and variation across studies on the characteristics of interest, we will aim to include these moderating variables in a single model. Analyses will be based on random effects, and will be undertaken using the metafor package in R (Viechtbauer, 2010). If there are insufficient number of effect sizes included in the review to undertake meta‐regression, we will attempt single characteristics sub‐group analysis. Any conclusions drawn from the meta‐regression analysis will be cautious and exploratory given that these relationships are observational in nature and likely based on a small number of effects.

#### 3.3.17 Sensitivity analysis

We will undertake sensitivity analysis to determine the effect on our overall findings of:

• Non‐randomised studies
• Studies with effect sizes which have been converted from binary to continuous (or vice versa)
• Studies classified as low confidence in findings

Sensitivity analyses will be undertaken by repeating the meta‐analysis, omitting in turn each of the groups of studies described above, to determine their effect on overall findings.

## CONTRIBUTIONS OF AUTHORS

- Chris O'Leary is the lead reviewer and is responsible for the overall delivery of the review. He is a public policy specialist, with a specific expertise in providing evidence based advice to policy makers, particularly in relation to interventions designed to prevent and reduce homelessness. He sits on the Manchester Homelessness Partnership, a collaboration between statutory and voluntary agencies in Manchester aimed at reducing homelessness, and improving and quality and effectiveness of homelessness services. His published research on homelessness includes empirical research and evidence reviews. His article reviewing the evidence around homelessness and recidivism (O'Leary, 2013) is rated by Altmetrics as in the top 25% of all research for policy impact, and his 2018 research on homelessness from the private rented sector in England was launched in the UK House of Commons by two All Party Parliamentary groups of Members of Parliament.
- Rob Ralphs is a substance use specialist with over 20 years' experience in conducting research on substance users, with a particular focus on drug treatment and policy. Since 2016, he has been the principal investigator on seven research projects that have focused on homelessness, substance use, service development and service user engagement. Through this portfolio of research, he has developed unrivalled experience and understanding of contemporary substance use trends and the challenges of engaging people with lived experience of homelessness into treatment. His recent 2020 paper on the motivations of synthetic cannabinoid use amongst people with lived experience of homelessness and the policy and practice responses is one of the most read articles in the leading international journal *Addiction, Research and Theory*. His current research focuses on the impact of COVID‐19 on access to drugs and emerging drug trends amongst those experiencing homeless in Greater Manchester. He currently sits on a number of advisory roles including: the Advisory Council for the Misuse of Drugs (ACMD) Novel Benzodiazepines and New Psychoactive Substances Monitoring Committee; the Greater Manchester Drug Alert Panel; the Manchester Professional Advisory Group for Drugs and Alcohol; the Manchester Needle and Syringe Provision Steering Group; the Greater Manchester Combined Authority COVID‐19 Homeless Hotel Substance Use Harm Reduction Advisory Group.
- Andrew Smith was responsible for the day to day operation of the review until February 2022. Jennifer Stevenson was responsible for the day to day operation from February 2022 and will be responsible for the meta‐analysis. This review will be supported by specialist statistical methods input from Zsolt Kiss alongside research support from senior research assistant Jordan Harrison and Harry Armitage.

## DECLARATIONS OF INTEREST

No conflicts of interest.

## PLANS FOR UPDATING THIS REVIEW

Dependant on additional funding.

## SOURCES OF SUPPORT

Internal sources

• None, Other

None provided

External sources

• Centre for Homelessness Impact, UK

Funding for systematic review

## Supporting information

Supplementary information.

## Footnotes

^1^

https://www.statsdirect.com/help/meta_analysis/heterogeneity.htm

^2^
Level descriptions taken from https://whatworksgrowth.org/resources/the-scientific-maryland-scale/
