# Supplementary material for: PROTOCOL: The effectiveness of abstinence‐based and harm reduction‐based interventions in reducing problematic substance use in adults who are experiencing severe and multiple disadvantage homelessness: A systematic review and meta‐analysis
Source: Campbell Syst Rev. 2022 Jul 7;18(3):e1246. doi: 10.1002/cl2.1246 (PMC9261927; doi:10.1002/cl2.1246)
Supplement: Supplementary file 1 — Supplementary information. [file CL2-18-e1246-s001.docx]

# Appendices

## 1 Appendix A: Search details from White et al., (2019): PROTOCOL: Studies of the effectiveness of interventions to improve the welfare of those affected by, and at risk of, homelessness in high‐income countries: An evidence and gap map

**Databases**:

1. Academic databases

• Econlit

• The National Bureau of Economic Research (NBER)

• Social Science Research Network (SSRN)

• International Bibliography of Social Sciences (IBSS)

• Applied Social Sciences Index and Abstracts (ASSIA)

• Social Service Abstract

• Embase

• PubMed

• PsycINFO

• MEDLINE

• WHO’s Global Health Library

• CABI’s Global Health

• ERIC

• CINHAL

• SCOPUS

• Web of Science

• EPPI Centre Evaluation Database of Education Research

2. Evidence and Gap Map Database

• 3ie Evidence and Gap Map Repository

• Global Evidence Mapping Initiative

• Evidence‐Based Synthesis Program (Department of Veteran

Affairs)

3. Systematic review databases

• Swedish Agency for Health Technology Assessment and

Assessment of Social Services

• Collaboration for Environmental Evidence

• Cochrane

• Cochrane

• Campbell

• 3ie Systematic Review Database

• Research for Development

• Epistemonikos

4. Trials registries

AEA Social Science RCT Registry https://www.socialscience

registry.org/.

**Grey literature and websites:**

Homeless Hub https://www.homelesshub.ca/

European observatory on homelessness https://www.feantsaresearch.org/en/publications

United State interagency council on homelessness http://www.usich.gov/

EThOS http://ethos.bl.uk/Home.do

WHO ICTRP http://apps.who.int/trialsearch/

Focus on Prevention http://www.preventionfocus.net/

Social Policy and Practice http://www.spandp.net/

10,000 home campaigns https://en.wikipedia.org/wiki/100,000_Homes_Campaign

Anti‐poverty committee https://en.wikipedia.org/wiki/Anti‐Poverty_Committee

Back on my feet https://en.wikipedia.org/wiki/Back_on_My_Feet_(non‐profit_organization)

Feantsa https://www.feantsa.org/

National Coalition Homeless https://nationalhomeless.org/

Homelessness Australia https://www.homelessnessaustralia.org.au/

Mission Australia https://www.missionaustralia.com.au/publications/position‐statements/homelessness

National Alliance to end homelessness https://endhomelessness.org/

Institute of global homelessness https://www.ighomelessness.org/

Homelessness link https://www.homeless.org.uk/

Crisis https://www.crisis.org.uk/about‐us/how‐we‐work/

Housing first https://housingfirsteurope.eu/about‐the‐hub/

Canadian Alliance to end homelessness https://housingfirsteurope.eu/about‐the‐hub/

Social work and policy institutes http://www.socialworkpolicy.org/research/homelessness.html

Association of housing advice services https://www.ahas.org.uk/

Centre point <https://centrepoint.org.uk/>

Homelessness trust funds https://housingtrustfundproject.org/htf‐elements/homeless‐trust‐funds/

Meliville charitable trust https://melvilletrust.org/category/resourcesreports/

Conrad H Hilton foundation https://www.hiltonfoundation.org/priorities/homelessness#resources

Abt Associates https://www.abtassociates.com/

Mathematica https://www.mathematica‐mpr.com/

American Institutes of Research https://www.air.org/

Rand https://www.rand.org/

MDRC https://www.mdrc.org/

**Additional searches using Google and Google Scholar.**

**SAMPLE SEARCH STRING**

Search string/keywords (for ovid medline platform)

**Study design key words**

– (“quasi experiment*” or quasi‐experiment* or “random* control*

trial*” or “random* trial*” or RCT or (random* adj3 allocat*) or

matching or “propensity score” or PSM or “regression discontinuity”

or “discontinuous design” or RDD or “difference in difference*” or

difference‐in‐difference* or “diff in diff” or “case control” or cohort or

“propensity weighted” or propensity‐weighted or “interrupted time

series” or (before adj5 after) or (pre adj5 post) or ((pretest or pre

test) and (posttest or post test)) or “research synthesis” or “scoping

review” or “rapid evidence assessment” or “systematic literature

review” or “Systematic review” or “Meta‐analy*” or Metaanaly* or

“meta analy*” or “Control* evaluation” or “Control treatment” or

“instrumental variable*” or heckman or IV or (quantitative or

“comparison group*” or counterfactual or “counter factual” or

counter‐factual or experiment*) adj3 (design or study or analysis))

or QED or evaluation).ti,ab,kw

– OR

– clinical trial/or clinical trial, phase i/or clinical trial, phase ii/or clinical

trial, phase iii/or clinical trial, phase iv/or controlled clinical trial/or

randomised controlled trial/or pragmatic clinical trial/

– controlled clinical trials as topic/or non‐randomised controlled

trials as topic/or randomised controlled trials as topic/or

pragmatic clinical trials as topic/or case‐control studies/or

retrospective studies/or controlled before‐after studies/or interrupted

time series analysis/or random allocation/or cohort

studies/or follow‐up studies/or longitudinal studies/or prospective

studies/or retrospective studies/or propensity score/or regression

analysis/or evaluation studies/or matched‐pair analysis

– (“quasi experiment*” or quasi‐experiment* or “random* control* trial*”

or “random* trial*” or RCT or (random* adj3 allocat*) or matching or

“propensity score” or PSM or “regression discontinuity” or “discontinuous

design” or RDD or “difference in difference*” or difference‐indifference*

or “diff in diff” or “case control” or cohort or “propensity

weighted” or propensity‐weighted or “interrupted time series” or

(before adj5 after) or (pre adj5 post) or ((pretest or pre test) and

(posttest or post test)) or “research synthesis” or “scoping review” or

“rapid evidence assessment” or “systematic literature review” or

“Systematic review” or “Meta‐analy*” or Metaanaly* or “meta analy*”

or “Control* evaluation” or “Control treatment” or “instrumental

variable*” or heckman or IV or ((quantitative or “comparison group*”

or counterfactual or “counter factual” or counter‐factual or experiment*)

adj3 (design or study or analysis)) or QED).ti,ab,kw.

(“meta regression” or “meta synth*” or “meta‐synth*” or “meta analy*”

or “metaanaly*” or “meta‐analy*” or “metanaly*” or “metaregression” or

“metaregression” or “methodologic* overview” or “pool* analys*” or “pool*

data” or “quantitative* overview” or “research integration”).ti,ab,sh.

OR

(review adj3 (effectiveness or effects or systemat* or synth* or

integrat* or map* or methodologic* or quantitative or evidence or

literature)).ti,ab,sh.

**Homelessness keywords**

– homeless persons/or homeless youth

– (evict* or homeless* or "housing excl*" or “residential stability”

or ((street* or private or improvised or shelter* or emergency or

temporary or insecure or overcrowded or precarious or stable or

marginal*) adj3 (dwell* or house* or housing or accommodation))

or (street adj3 (life or living or lives or youth* or child* or people or

person*)) or runaway* or “Run away from home” or “Running away”

or “Ran away” or “Going missing” or “Bag lady” or Houseless* or

Unhoused or “without a roof” or Roofless or (rough adj3 sleep*) or

Destitut* or “Skid row*” or “sleepers out”).ti,ab,kw.

– (“Housing first” or “Pathways to Housing” or “Homeless Veterans

Reintegration Program” or “Access to Community Care and Effective

Services and Supports” or 'Support* Housing Program” or “Housing

and Urban Development–Veterans Affairs Supported Housing program”

or “HUD‐VASH” or “Sober Transitional Housing and Employment

Project” or “sober house placement*” or “Housing ladders” or

“Staircase housing” or “low threshold housing” or “Critical Time

Intervention”).ti,ab,kw

## 2 Appendix B: Data extraction form

| **Bibliographic Information** | |
| --- | --- |
| Authors | FREE TEXT |
| Title | FREE TEXT |
| Year | FREE TEXT |
| Journal | FREE TEXT |
| Type | 1. Journal Article 2. Dissertation/Thesis 3. Report 4. Other |
| Abstract | FREE TEXT |
| Country | 1. United States of America 2. Australia 3. Canada 4. Denmark 5. United Kingdom 6. Netherlands 7. Ireland 8. France 9. South Korea |
| Age | 1. Mean age 2. Age range |
| Complexity of needs (other challenges participants face, other than homelessness and substance misuse) | 1. Discharge from health services 2. Mental illness 3. Complex needs/dual diagnosis 4. Veteran/Ex-services 5. Drug and alcohol issues 6. Survivor domestic violence/abuse 7. Migrant 8. Ex-prisoner 9. Indigenous persons 10. Care leaver |
| Gender *% (actual number)* | 1. Male 2. Female 3. Male and female |
| **Typology classification** | 1. Abstinence-based 2. Harm reduction 3. Abstinence-based and harm reduction |
| **Intervention classification** | 1. Psychosocial intervention 2. Treatment through medication 3. Non-medication intervention |
| **Comparison** | 1. Other intervention 2. Treatment as usual 3. Waitlist |
| Comparison intervention | FREE TEXT |
| **Outcomes measures** | FREE TEXT (e.g. substance abuse, recidivism, employment |
| Substance misuse outcome measure | FREE TEXT |
| Total sample size | FREE TEXT |
| Size of treated group | FREE TEXT |
| Size of control group | FREE TEXT |
| Data to calculate effect size | As required – based on ultimate choice of effect size |
| **Campbell’s Critical Appraisal Tool for Primary Studies** | |
| Study design at end of intervention (Potential confounders taken into account) | 1. DiD with matching, PSM 2. RCT, RDD, ITT, instrumental variable 3. Other |
| Study design, longest follow up if applicable (Potential confounders taken into account) | 1. DiD with matching, PSM 2. RCT, RDD, ITT, Instrumental variable 3. Other |
| Masking or blinding | 1. Unblinded or no mention of blinding 2. Any blinding or any mention of blinding |
| Power calculation | 1. Any mention of power calculations as basis for sample size 2. No mention of power calculation |
| Losses to follow up are presented and acceptable, End of intervention | 1. No mention of attrition 2. Attrition not reported or below accepted level 3. Overall and differential attrition close to accepted level 4. Overall and differential attrition within accepted level 5. N/A (ex-post study) |
| Losses to follow up are presented and acceptable (Longest follow up, if applicable | 1. No mentioned of attrition 2. Attrition not reported or below accepted level 3. Overall and differential attrition close to accepted level 4. Overall and differential attrition within accepted level 5. N/A (ex-post study) |
| Definition of intervention are clearly defined | 1. Brief description of intervention 2. Intervention clearly and fully described 3. Intervention named but not described, or not named |
| Outcome measures are clearly defined and reliable | 1. Brief description of outcome 2. Outcome measure clearly and fully described 3. Outcome named but not described, nor not named |
| Baseline balance (N/A for before versus after) | 1. Imbalance on 2 or less measures 2. Imbalance on no more than 5 measures 3. No baseline balance test, or imbalance on 5+ measures |
| Overall confidence in primary study | 1. Low 2. Medium 3. High |

## 3 Appendix C: Campbell’s Critical Appraisal Tool for Primary Studies (White et al., 2020)

“The tool for primary studies has seven items which relate to 1) study design, 2) blinding, 3) power calculations, 4) attrition, 5) description of the intervention, 6) outcome definition and 7) baseline balance. A fuller description of these items is given in the technical appendix. Each of these seven items is rated as implying high, medium or low confidence in study findings. Overall quality is assessed using the ‘weakest link in the chain’ principle: our confidence in study findings can only be as high as the lowest rating given to any of the critical items (which are numbers 1, 4, 6 and 7).” (White et al., 2020, p.31)

| Item | | Point in time (where applicable) | Rating |
| --- | --- | --- | --- |
| 1a | Study design (Potential confounders taken into account) | End of intervention | High confidence: RCT, RDD, ITT, instrumental variable  Medium confidence: DiD with matching, PSM  Low confidence: other matching |
| 1b | Study design (Potential confounders taken into account) | Longest follow up (if applicable) | Study design may change at post endline follow up, usually loss of RCT as control becomes treated. Same codes as 1a |
| 2 | Masking or blinding (RCTs only) |  | High confidence: any blinding or any mention of blinding  Medium confidence: no blinding  **Low confidence is not used for this item** |
| 3 | Power calculations are reported |  | High confidence: any mention of power calculations as basis for sample size  Medium confidence: no mention of power calculations  **Low confidence is not used for this item** |
| 4a | Losses to follow up are presented and acceptable* | End of intervention | High: attrition within IES bounds  Medium: attrition within IES liberal bounds  Low: attrition not reported or attrition outside IES bounds  N/A for ex post studies |
| 4b | Losses to follow up are presented and acceptable* | Longest follow up (if applicable) | High: attrition within IES bounds  Medium: attrition close to IES bounds  Low: attrition not reported or attrition outside IES bounds  N/A for ex post studies |
| 5 | Intervention if clearly defined |  | High confidence: intervention clearly and fully described  Medium confidence: brief description of intervention  Low confidence: intervention named but not described, or not named |
| 6 | Outcome measures are clearly defined and reliable |  | High confidence: outcome measure clearly and fully described, preferably with reference to validation  Medium confidence: brief description of outcome  Low confidence: outcome named but not described |
| 7 | Baseline balance (N.A. for before versus after) |  | High confidence: RCT or baseline balance report and satisfactory (imbalance on 2 or less measures)  Medium confidence: Imbalance on no more than 5 measures  Low confidence: Baseline balance not reported, or reported and lack of balance on more than 5 measures |
|  |  |  |  |
|  | **Overall confidence in study findings** | **End of intervention** | **Lowest rating across items 1a, 4a, 6 and 7** |
|  | **Overall confidence in study findings** | **Longest follow up (if applicable)** | **Lowest rating across items 1b, 4b, 6 and 7 (N/A if 1b and 4b N/A)** |
|  | * See table 1 <https://homvee.acf.hhs.gov/sites/default/files/2019-06/HomVEE-Attrition-White_Paper-7-2015.pdf> | | |

## 4 Appendix D:  Glossary of interventions

***Abstinence based day programmes*** are typically structured day treatment programmes for people with drug and/or alcohol problems. They may offer a range of abstinence-based treatment options which integrate 12-Step Recovery Programmes, CBT and other appropriate therapies. They may also include personally rewarding structured training, counselling and self-help.

***Agonist pharmacotherapy /blockers*** are drugs that bind to and activates receptors. *Agonist* treatments currently include methadone (which is the mainstay of UK *agonist* treatment services), although in some countries or localities prescribed *heroin*, dihydrocodeine and LAAM (levo-methadol) are used.

***Assertive Community Therapy (ACT)*** was originally developed for patients with severe mental illness, providing personalized, high intensity, holistic and integrated multidisciplinary community care services.

***Assertive Outreach*** models recognise that for some drug and alcohol users, their chaotic lifestyle and motivation may lead to non-engagement or missed appointments. Drug and alcohol assertive outreach services aim to increase engagement and improve re-engagement of clients dropping out treatment services. Assertive outreach services will work with clients in a variety of settings to ensure maximum engagement and re-engagement with drug and alcohol treatment services. This may include on the streets or in day centres, hostels and supported accommodation.

***Behaviour Couples Therapy (BCT)*** is a form of behaviour therapy originally developed to treat depression, that addresses elements of the couple’s relationship that are known to have a direct effect on the incidence of depression. A key component of this therapy is to focus on improving communication difficulties within the relationship. BCT will aim to specifically improve the overall quality of the relationship.

***Cognitive-Behavioural Therapy (CBT)*** is a therapeutic approach that seeks to modify negative or self-defeating thoughts and behaviour. CBT is aimed at both thought and behaviour change—that is, coping by thinking differently and coping by acting differently. Cognitive behavioural therapy is a form of psychotherapy that teaches people strategies to identify and correct problematic associations among thoughts, emotions, and behaviours to enhance self-control and reduce drug use.

***Contingency Management (CM)*** is an approach to treatment that maintains that the form or frequency of behaviour can be altered through a planned and organized system of positive and negative consequences. This typically includes regular testing and requirements for treatment engagement. CM assumes that neurobiological and environmental factors influence substance use behaviours and that the consistent application of reinforcing environmental consequences can change these behaviours.

***Drug Consumption Rooms (DCRs)*** allow people to take drugs in clean, clinically supervised spaces, where they can also access advice and support. In some cases, these also facilitate non-injecting drug use. which have been shown to reduce overdoses and transmission of HIV.

***Detoxification*** involves a clearing of toxins from the body. In substance use treatment, it is a term used to describe the medical and biopsychosocial procedure that assists a person who is dependent on one or more substances to withdraw from dependence on all substances of abuse.

***Eye Movement Desensitization and Reprocessing (EMDR)*** is a psychotherapy treatment that was originally designed to alleviate the distress associated with traumatic memories. EMDR therapy facilitates the accessing and processing of traumatic memories and other adverse life experience to bring these to an adaptive resolution. During EMDR therapy the client attends to emotionally disturbing material in brief sequential doses while simultaneously focusing on an external stimulus. Therapist directed lateral eye movements are the most commonly used external stimulus but a variety of other stimuli including hand-tapping and audio stimulation are often used.

***Group work*** in the context of substance use treatment can involve a variety of group treatment models to meet client needs during the multiphase process of recovery. This may include skills development groups, cognitive–behavioural/problem solving groups, or support groups.

***Harm reduction-based day centres*** provide support to help adults reduce the harm drugs and alcohol can cause and often provide outreach and in-reach support.

***Harm reduction psychotherapy*** draws on the concept of harm reduction within a psychotherapeutic approach that integrates cognitive and behavioural interventions with a psychodynamic understanding of substance use as personally meaningful.

***Heroin Assisted Therapy (HAT)*** is the prescription of heroin (diamorphine). It is typically used for people who do not respond to OST. This treatment has been shown to improve health outcomes in some of the most dependent of those people who inject drugs.

***Motivational Enhancement Therapy (MET)*** is a counselling approach that helps individuals resolve their ambivalence about engaging in treatment and stopping their drug use. This approach aims to evoke rapid and internally motivated change, rather than guide the patient stepwise through the recovery process.

***Motivational Interviewing (MI)*** is a client-centred, directive method for enhancing intrinsic motivation to change by exploring and resolving ambivalence.

***Naloxone*** is widely used as an effective opioid overdose reversal agent. It is an opioid antagonist that effectively blocks the effects of opioids (heroin, methadone, opium, codeine, morphine and buprenorphine) if they are used.

***Needle exchanges*** are community-based prevention programs that can provide a range of services, including linkage to substance use disorder treatment; access to and disposal of sterile syringes and injection equipment; and vaccination, testing for blood borne viruses, and linkage to care and treatment for infectious diseases.

***NX provision*** is an abbreviated term commonly used to refer to needle exchange services that provide and facilitate the return of used needles and syringes that support a reduction in transmission of blood-borne viruses through the distribution of injecting drug use equipment.

***Opioid Substitution Treatment (OST)*** is the prescribing of a replacement drug such as methadone or buprenorphine. This is one of the most evidence-based treatments on offer to reduce illicit opiate use, overdoses and transmission of blood borne viruses such as HIV and viral hepatitis.

***Opioid Agonist Therapy (OAT)*** is a treatment for opioid use disorder that uses opioid agonists, such as methadone or buprenorphine, which bind to the same receptors in the brain activated by the drug of misuse, but in a safer and more controlled manner. These medications reduce withdrawal symptoms and cravings.

***Prescripting to prevent relapse*** A number of relapse prevention medications exist. These include *Naltrexone*, an opioid antagonist that reduces cravings and decreases the pleasure of alcohol consumption. These medications are used in both the early stages of treatment and for long-term recovery.

***The Reduction and Motivation Programme (RAMP)*** provides a safe environment for those in active addiction to explore their dependency and its impact on themselves and others and the life changes needed to gain recovery from substances. It is typically run as a 12-week programme with two sessions per week.

***Rapid prescribing*** means that once clients have been referred, they can obtain their prescription (e.g. methadone or buprenorphine) within a short space of time e.g. 24 hours, compared to typically waiting several weeks.

***Residential rehabilitation*** is typically based on ***‘therapeutic community’*** treatment for substance users. These abstinence-based programmes are more intensive and expensive interventions.

***Safe Injecting Facilities*** are sometimes known as *overdose prevention centres* or *drug consumption rooms*. They are places where people are allowed to inject illegal drugs in hygienic conditions in the supportive presence of medical staff and peer workers.

***Self-help/mutual aid support service*** are most commonly associated with 12-step, Alcoholics Anonymous (AA), Narcotics Anonymous (NA), Cocaine Anonymous (CA). However, many other models are available such as Smart Recovery. They may serve as the primary or only source of behaviour change for many, or as aides to formal treatment, or as a form of continuing care and community support following exit from treatment. These groups are highly accessible and free in communities, and serve as important and readily available resources in substance abuse recovery.

***Testing for BBVs*** typically involves testing for blood borne viruses that are more prevalent amongst injecting drug users such as hepatitis C and HIV. Testing facilities may also provide vaccinations including for Hep, A, B and C. These services are commonly offered in needle exchanges and by drug treatment services.

***Therapeutic communities (TCs)*** refer to a consciously designed social environment or residential treatment setting in which the social and group process is harnessed with therapeutic intent. The TC promotes abstinence from alcohol and illicit drug use, and seeks to decrease antisocial behaviour and to effect a global change in lifestyle, including attitudes and values. The TC employs the community itself as the agent of healing. The TC views drug abuse as a disorder of the whole person, reflecting problems in conduct, attitudes, moods, values, and emotional management. Treatment focuses on drug abstinence, coupled with social and psychological change that requires a multidimensional effort involving intensive mutual self-help typically in a residential setting.

***Trauma therapies*** is most commonly associated with Eye Movement Desensitization and Reprocessing (EDMR).

***Treatment through medication*** refers to the prescribing of substitute medication or blockers such as methadone, buprenorphine and naltrexone.
